# Mass testing to support sustained containment of COVID-19

**DOI:** 10.7189/jogh.11.03114

**Published:** 2021-10-02

**Authors:** Weibin Cheng, Na Zhao, Yiru Qin, Junzhang Tian, Yongshun Huang

**Affiliations:** 1 Institute for Healthcare Artificial Intelligence Application, Guangdong Second Provincial General Hospital, Guangzhou, China; 2 School of Data Science, City University of Hong Kong, Hong Kong SAR of China; 3 Department of risk assessment, Guangdong Province Hospital for Occupational Disease Prevention and Treatment, Guangzhou, China; 4 Guangdong Provincial Key Laboratory of Occupational Disease Prevention and Treatment, Guangdong Province Hospital for Occupational Disease Prevention and Treatment, Guangzhou, China

Mass testing for covid-19 has great potential, if delivered to the right people at the right time with the right ongoing action, to bring benefit for containing the epidemic and to support the government in reopening economic activities [1-3]. As noted in a recent Nature Medicine Correspondence[4], China has implemented mass, community-wide testing actions which successfully helped to support sustained containment of covid-19. For instance, during the outbreak in Guangzhou, 18.7 million people were tested within three days [5]. Although the use of mass testing has not reached the agreement by all experts [6,7], the experience from China display its great value for covid-19 control and prevention. In this viewpoint, we describe six key elements of effective implementation of mass testing for covid-19 in the China context.

First, confine. Time, areas, and targets of mass testing for COVID-19 should be defined based on the evidence of epidemiological investigations, by which to identify the route of transmission, sort out the range of close contacts, and divide risk areas (low, moderate, or high risk of transmission). The window period of implementation mass covid-19 testing is at the early stage of community transmission. Mass gathering testing events are highly recommended to conduct in low and moderate risk areas. Although it is not strictly mandatory, the local residents highly participated in this testing program. Close contacts of covid-19 patient/s should not be encouraged to participate in the mass testing to lower the risk of onward transmission during such mass gathering events.

Second, training and monitoring. Large-scale community-based covid-19 testing requires a lot of manpower, many of whom may not have been previously trained and work in an intensified situation. Though medical staff may have been trained for the nasal pharyngeal swab or pharyngeal swab sampling, intensified training with drills and assessments are needed that follow national guidelines to ensure standard and uniformity of sample collection by all staff. All staff need to comply with biosafety requirements and be capable of putting on and taking off personal protective equipment (PPE) correctly. Sample takers should be self-health monitored for 14 days after fieldwork. If there are no positive cases among the tested population, a nucleic acid test for SRAS-CoV-2 RNA will be carried out for all staff on the presupposed day (the medium incubation period, for COVID-19 Delta Variant B.1.617.2 is 4 days [6]). If there is a positive case, four further PCR tests will be carried out for all sample takers on days 1st, 3rd, 7th, and 14th.

**Figure:**
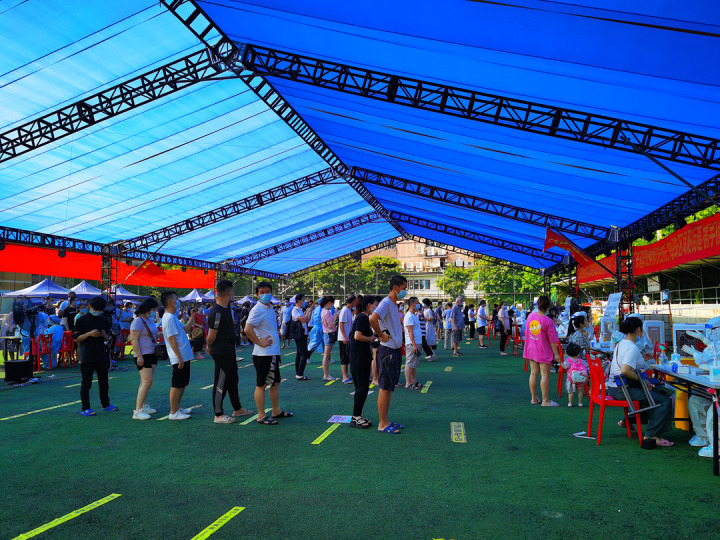
Photo: Local residents participated in the mass testing program in Guangzhou, China (from the collection of Huang Jinjiang, used with permission).

Third, testing capacity. The success of mass testing for covid-19 largely depended on the laboratory network infrastructure capacity. We adapted the 5:1 or 10:1 pooled sampling approach by combining five or ten specimens and test the combination for SARS-CoV-2 RNA to increase the efficiency and reduce the cost. If the combination tests returned a positive result, people whose samples were combined are tested separately to identify the infected individual. Two air-inflated COVID-19 test laboratories, namely Huo-Yan Laboratory [7] and Falcon laboratory [8], were built in one day to meet the surge of testing demands in Guangzhou. The Huo-yan laboratory has a testing capacity of 150 000 samples per day, the highest in the country. By using the 10 in 1 testing method, the testing capability can increase to 1.5 million samples per day.

Fourth, communication. Constantly communicate with the public with the updates of epidemic information is important, this encourages the public to take necessary control measures and to get involved in mass testing[9]. The local authority should issue a public notice to clarify the purpose of mass testing and give out detailed information on who should get tested, when and where the testing event will be held, and what are the precautions? Once test takers arrive at the mass testing center, they fully informed and requested to provide informed consent to avoid panic, misunderstanding, and unnecessary disputes.

Fifth, infection control. On-site cross infection control should be the top priority of implementing the mass covid-19 testing program. An infection control plan is mandatory for monitoring the sampling process and the use of PPE. An open and well-ventilated location as a centralized sample collection site should be selected. The site can be divided into waiting areas, collection areas, buffer zones, and temporary isolation areas, with clear instructions on the direction of flow for people. Individuals are to be separated by a one-meter distance to reduce the risk of cross-infection. All people must wear a mask and are refrained to talk in the waiting area. All sample takers are required to self-monitor for 14 days after fieldwork. Nucleic acid test for SRAS-CoV-2 RNA should be carried out for all staff according to the medium incubation period (for COVID-19 Delta Variant B.1.617.2 is 4 days [6]).

Sixth, swift registration. Information registry is a key link in the rapid mass testing process. Based on the favorable conditions of the high penetration rate of smartphones in China, a distributed information registration platform was developed. Test takers use their smartphones to scan the QR code of the platform to complete registration. Information registered is kept to a minimum by including only name, gender, ID number, and contact number. Other information such as the location, date, and time are automatically entered to form a bar code to be verified. Staff in charge of registration use a smartphone to scan the barcode information generated by the test takers to complete the verification. They allocate 10 or 5 test takers as a group to combine their samples in one tube. Considering subjects without smartphones, the staff should archive them through the paper registration form before submitting the specimen for testing, so that the subject can be traced back in time.

In facing of the emerging covid-19 variant of concerns, scaling up population vaccination is no time to delay. Enforcing the containment strategies at the early stage may become a new normalcy covid-19 containment strategy in pre-and even post-herd immunity period. The epidemiological investigations, lockdown of key areas, and mass testing ensure timeliness of early case detection and interruption of local outbreaks.
